# Operando Characterizations of Light-Induced Junction Evolution in Perovskite Solar Cells

**DOI:** 10.1021/acsami.2c22801

**Published:** 2023-04-18

**Authors:** Chuanxiao Xiao, Yaxin Zhai, Zhaoning Song, Kang Wang, Changlei Wang, Chun-Sheng Jiang, Matthew C. Beard, Yanfa Yan, Mowafak Al-Jassim

**Affiliations:** † National Renewable Energy Laboratory (NREL), Golden, Colorado 80401, United States; ‡ The University of Toledo, Toledo, Ohio 43606, United States; § Key Laboratory of Low-Dimensional Quantum Structures and Quantum Control of Ministry of Education, Department of Physics, Hunan Normal University, Changsha 410081, China

**Keywords:** perovskite solar cell, light soak, electric field, interfacial recombination, junction, operation mechanism, operando characterizations

## Abstract

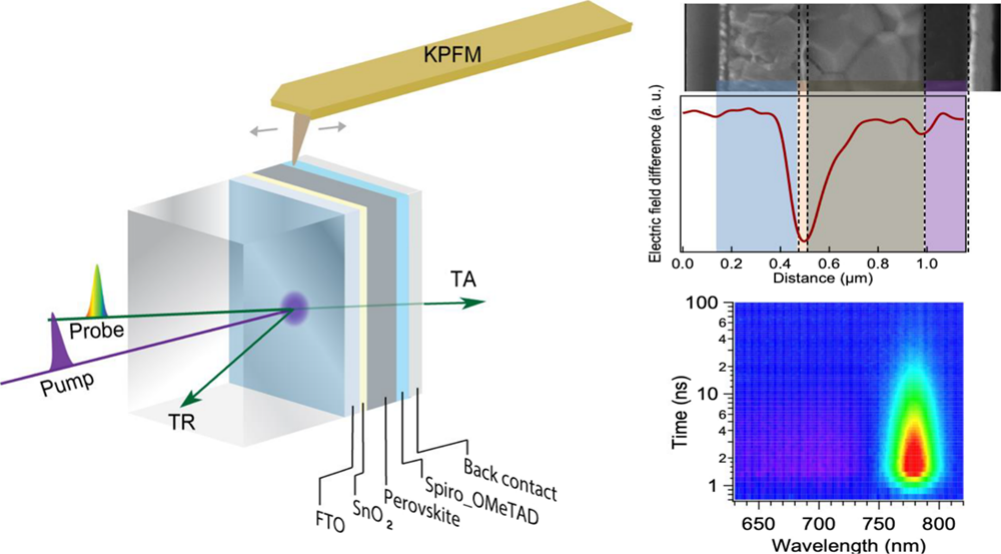

Light-induced performance changes in metal halide perovskite solar cells (PSCs) have been studied intensively over the last decade, but little is known about the variation in microscopic optoelectronic properties of the perovskite heterojunctions in a completed device during operation. Here, we combine Kelvin probe force microscopy and transient reflection spectroscopy techniques to spatially resolve the evolution of junction properties during the operation of metal-halide PSCs and study the light-soaking effect. Our analysis showed a rise of an electric field at the hole-transport layer side, convoluted with a more reduced interfacial recombination rate at the electron-transport layer side in the PSCs with an n–i–p structure. The junction evolution is attributed to the effects of ion migration and self-poling by built-in voltage. Device performances are correlated with the changes of electrostatic potential distribution and interfacial carrier dynamics. Our results demonstrate a new route for studying the complex operation mechanism in PSCs.

## Introduction

Perovskite solar cell (PSC) technology has advanced rapidly in recent years^1−8^ and attracted enormous attention from both the research community and industrial manufacturers. Thanks to global efforts, the certified power conversion efficiency has reached a remarkable 25.7%, comparable with the best record of single-crystal silicon solar cells and surpassed that of other polycrystalline thin-film photovoltaics such as copper indium gallium selenide and cadmium telluride (CdTe).^9^ This outstanding device performance is owing to the extraordinary optoelectronic properties of metal-halide perovskite semiconductors, such as a strong light absorption coefficient, a high carrier mobility, and a long carrier lifetime.^5,10−13^ More importantly, the rapid development of PSCs is attributed to the facile solution-based printing and coating processes for perovskite deposition. The unique defect tolerance makes it relatively easy to synthesize high-crystallinity materials using non-vacuum-based low-temperature deposition approaches, enabling high-efficiency devices.^14−17^ Nowadays, it is pervasive to make >22% efficient PSCs on a regular basis in many laboratories all over the world. Meanwhile, the commercialization of the PSC technology is making good progress. With the common goal of achieving both high efficiency and stable PSCs, enabling these goals requires a comprehensive understanding of both the material properties and device physics as well as the interplay between them.

Metal-halide perovskite semiconductors are both highly and easily tunable, where deliberate tuning or unintentional small changes in composition could greatly impact their optoelectronic properties.^18−20^ Perovskite semiconductors can be slightly tuned to exhibit weakly p-type, weakly n-type, or intrinsic^21−24^ behavior due to a slight off-stoichiometry and/or ion migration due to the presence of halide vacancies. The metastable perovskite reportedly may change its optoelectronic properties during operation under illumination or even in the dark.^25−31^ Particularly, a variety of light-soaking effects have been observed, including light-induced lattice expansion,^32^ phase segregation,^33,34^ reversible defects,^35^ structural evolution,^36^ persistent photoconductivity,^37^ and performance degradation^38,39^ and enhancement.^40^

Light-soaking effects or light-induced cell performance changes have been observed and studied in inorganic photovoltaic semiconductors such as amorphous silicon and CdTe. For instance, in the most pronounced instance of amorphous silicon solar cells, light soaking causes the well-known Staebler–Wronski effect, resulting in the reduction of photoconductance and an increase of non-radiative recombination due to an increase in dangling bonds caused by a light-induced breaking of silicon–hydrogen bonds.^41−43^ In CdTe solar cells, copper ion migration from the back copper contact was found to be the main cause of photodegradation.^44,45^ However, in PSCs, light soaking could cause substantial changes of the intrinsic material as well as when incorporated into the device in a variety of ways, and their origins are still debated. The proposed mechanisms are ion migration that reduces defect densities,^27,28^ local polarization,^46,47^ change of photoconductance^29,48^ or light soaking enhanced electric field and charge accumulation when charge transport occurs between the perovskite semiconductor and the electrode interface, and finally reduced non-radiative recombination that can be induced within the perovskite bulk and at their surfaces and interfaces.^30,49^ Yet, little is known about the changes in the microscopic properties of perovskite heterojunctions and the evolution of these properties has rarely been characterized during device operation. A detailed understanding of the affected device operation mechanism, including charge-carrier separation, transport, and bulk/interface recombination, is lacking for PSCs.

**Figure 1:**
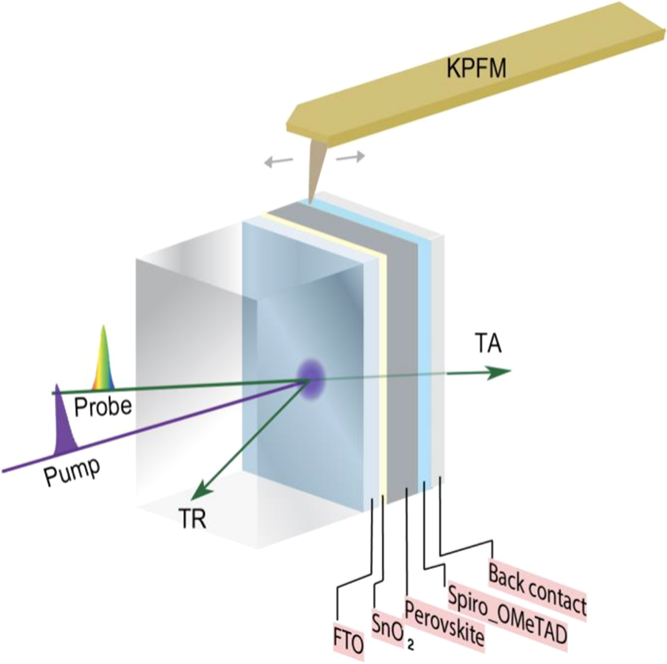
Experiment schematic. KPFM scans on the cleaved cross-sectional surface. Ultrafast spectroscopy measurements were in both TA and TR setups.

Here, we combine operando Kelvin probe force microscopy (KPFM) to scan the nanometer-scale electrostatic potential distribution and ultrafast spectroscopy to probe the interfacial carrier dynamics across PSCs to study the light-soaking effect. The experimental schematic is displayed in Figure 1. In a functioning device, KPFM scans the cross-sectional surface to profile and observe the differences in electric-potential (or electric-field) distribution; the operando KPFM results closely reflect the real case during current density–voltage measurements or cell operation. Femtosecond ultrafast spectroscopy has been operated in both transmissive and reflective geometry, in which the transient absorption (TA) measures the total carrier recombination process in the active layer while the transient reflection (TR) is more sensitive to the surface carrier dynamics (i.e., surface recombination and carrier diffusion away from the surface and into the bulk). The combination of these two techniques allows us to spatially resolve the electric-field distribution and capture interfacial carrier dynamics on a functioning solar cell, generating a more comprehensive picture of device operation. As a case study, we characterized a PSC with a standard architecture of fluorine-doped tin oxide (FTO) coated glass/SnO_2_/C_60_-SAM/ perovskite/Spiro-OMeTAD/Au, where SnO_2_/C_60_-SAM acts as the electron-transport layer (ETL), the perovskite absorber has a triple-cation composition of MA_0.63_FA_0.27_Cs_0.1_PbI_3_, and Spiro-OMeTAD works as the hole-transport layer (HTL), fabricated following a previous report.^50^ We measured the as-made cell and after light soaking and dark rest.

## Results and Discussion

**Figure 2:**
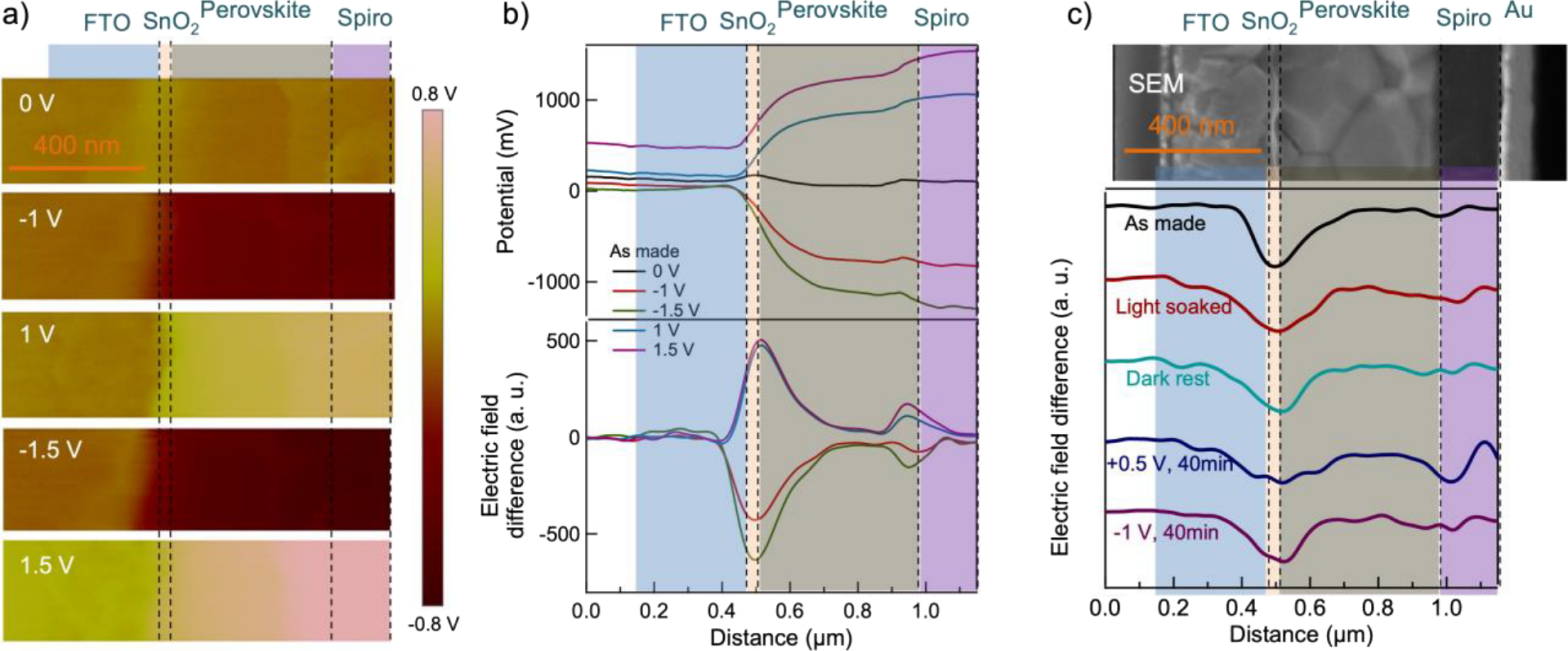
Operando characterization of electric potential profiling in a perovskite solar cell. (a) Potential images recorded at 0, −1, 1, −1.5, and 1.5 V bias voltages. (b) Top: potential profile average from images in (a), along the lateral direction; bottom: change in the electric field, calculated by taking the first derivatives of the potential difference than the 0 V profile. (c) Electric field difference profiles at −1 V aligned with the scanning electron microscopy image, where the device went through the process of (1) as-made, (2) light soaked, (3) after dark rest, (4) applied +0.5 V forward bias for 40 min, and (5) applied −1 V reverse bias for 40 min, showing the evolution of electric-field distribution.

In the KPFM measurements, we probe the electrostatic-potential distribution of a PSC by scanning the cross-sectional surface of the device. In our routine measurements for resolving the electric field distribution in a solar cell from KPFM surface potential measurement, various bias voltages are applied to the device while the surface potential is mapped and compared to the 0 V short-circuit condition. By applying a bias voltage, the junction characteristic can be assessed by measuring the voltage drop across the device stack. The voltage drop across the device is determined by the small current flow or the equivalent resistance of different layers and interfaces. During the KPFM scan, the atomic force microscope (AFM) topography and surface potential image were acquired simultaneously. We aligned the AFM images with potential images taken on the same location (Figure 2a). By averaging the lines in potential images, we obtained the potential profiles (Figure 2b, top). We then subtracted the 0 V profile from the biased profiles to get “potential difference” curves, where the effects of static surface charges can be eliminated. Finally, we numerically calculate the first derivative of the potential differences and plot the electric-field distribution relative to the metallurgical interfaces (Figure 2b, bottom). The as-made device showed that the main junction occurs at the ETL–perovskite interface and a smaller peak at the perovskite–HTL interface, while the electric field within the perovskite layer is close to zero, consistent with previous studies.^51,52^

We monitored the junction evolution after light soaking and dark rest (Figure 2c). The light comes from a halogen lamp with an intensity of ∼50 mW/cm^2^, and we did the measurements with in situ light soaking after 200 min illumination; we remeasured the cell after 20 h dark rest on the AFM stage. The light soaking specifically leads to a change in the junction properties. Interestingly, the electric field peak at the perovskite–HTL interface increases. The depletion regions (where there exists a non-zero electric field) at both the ETL–perovskite and perovskite–HTL junctions are slightly extended into the perovskite layer, making the device more like a conventional p–i–n structure with a depleted absorber layer.^53−55^ The stronger peak and wider depletion after light soaking may benefit charge collection of the photogenerated carriers. With an enhanced depletion width at the perovskite–HTL junction, charge collection can be easier with the help of the extended electric field. In this case, both the ETL–perovskite and perovskite–HTL interfaces change, unlike some of our previous studies that found that only one interface was modified.^51,52^ Because this measurement applies a voltage across the whole device architecture, both interfaces changed, hence, it is difficult to quantify which interface improves further or normalizes one interface to compare with others. While the ETL–perovskite peak intensity may seem to drop, the results do not imply a decrease of the ETL–perovskite junction. This is a limitation of the KPFM technique, where it relies on the potential drop applied to the device. More voltage drop has been re-distributed to the HTL–perovskite interface, and the electric field peak intensity reflects the competition of the ETL–perovskite and perovskite–HTL interfaces. The electric profiling results indicate perovskite–HTL interface has a more pronounced enhancement, possibly due to the rise of an electric field. After dark rest for 20 h, the ETL–perovskite junction returned to a state that is similar to the pristine condition, whereas the perovskite–HTL peak intensity decreased but was still larger than the as-made condition. These results suggest light soaking causes reversible junction evolution, and the device may have changed from the as-made condition.

We speculate that ion migration plays an important role during light soaking. There could be a light-induced self-poling effect, where the light causes migration/drift of charged interstitials (e.g., MA^+^, FA^+^, I^–^) or vacancies (V_MA_^–^, V_I_^+^) in the perovskite layer.^56−58^ In addition, extrinsic dopants, e.g., the Li^+^ ions, which are commonly used as a dopant to Spiro-OMeTAD, may also diffuse or drift into the perovskite layer.^23,59^ These ions may heal the defect/trap states at the interfaces, which leads to a reduced interface recombination rate and a better junction quality. In addition, the ions may change the effective doping density in the perovskite layer, particularly at the ETL–perovskite and perovskite–HTL interfaces. These modifications by mobile ions contribute to the junction evolution observed in Figure 2c. We also performed similar KPFM experiments on the same device under forward and reverse bias in the dark, the junction evolution trend is similar to the light soaking and dark rest, which confirms the effects of ion migration and self-poling by the built-in voltage. The detailed profiling results are shown in Figures S1–S4. We investigated ion redistribution by comparing 0 V potential profiles. However, we did not observe a clear trend in positive and negative ion migration (Figure S5). It is possible that the cross-sectional surface defect states may have changed after light soaking, which are good examples that the cross-sectional KPFM technique without bias voltages relies on surface potential and may lead to unreliable analysis.

**Figure 3:**
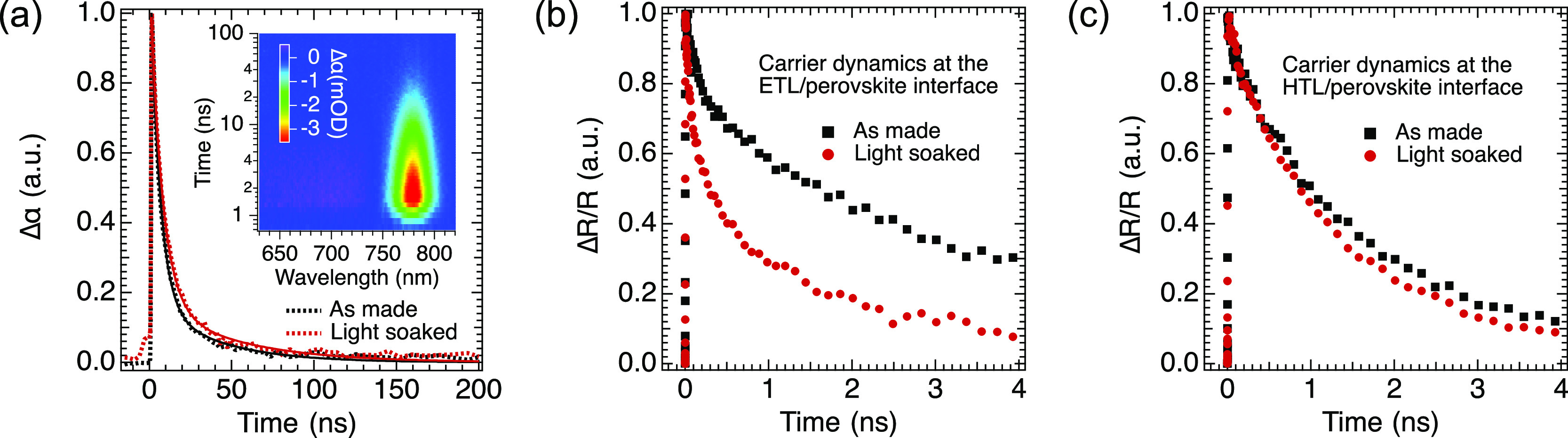
Ultrafast spectroscopy measurement of a perovskite solar cell. (a) Bulk carrier dynamics in the active layer. The inset shows the pseudo-color image of the TA spectra in the as-made device. Interface carrier dynamics at the (b) ETL–perovskite and (c) HTL–perovskite junctions. All measurements were performed on both as-made (black) and light-soaked (red) devices.

We used femtosecond ultrafast spectroscopy as a complementary technique to understand the junction evolution mechanism under light soaking. To separate the evolution of the ETL–perovskite and perovskite–HTL interfaces, we have performed the measurements on the devices in both transmissive and reflective geometry, see Figure 1. TA is operated in transmissive mode and measures the total carrier recombination process but is dominated by the bulk contributions in this configuration.^60^ In Figure 3a, we plot the nanosecond scale TA spectra in the devices before and after light soaking. The pseudo-color image of the TA spectra in Figure 3a inset shows similar features as that found in the pristine perovskite thin films indicating that we have selectively excited the perovskite layer and excluded impacts from the other layers.^61^ While the TA spectra at a specific delay time are not affected by the light soaking (Figure S6), the carrier lifetime obtained from the photobleaching dynamics has increased from 11.3 ± 1.4 ns in the as-made device to 14.6 ± 1.8 ns in the light-soaked one.

In thin films, the TA spectroscopy measures the total carrier lifetime (τ_T_) that is determined by both carrier lifetime in bulk (τ_B_) and the surface carrier dynamics with the following relation:
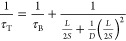
1in which *L* is the film thickness, and *S* and *D* are the two main parameters that govern the surface carrier dynamics, namely, surface recombination velocity and the carrier diffusion coefficient, respectively. When the film thickness is large (e.g., >300 nm in the case of the perovskite film), the bulk lifetime becomes the dominant factor in determining the total lifetime.^62^ The polycrystalline perovskite thin films used in high-efficiency solar cells have been reported to have total carrier lifetimes in the range of 100 ns measured by TA.^61^ However, our TA measurements on devices have indicated a total lifetime of approximately 10 ns, as indicated in Figure 3a. This suggests that surface recombination and carrier diffusion/extraction are limiting the perovskite device’s total lifetime.

Figure 3a indicates that the light-soaking elevates the total carrier lifetime from 11.3 ± 1.4 ns in the as-made device to 14.6 ± 1.8 ns. Based on eq 1, there are two possible mechanisms involved. First, the bulk carrier lifetime may increase, providing additional evidence that the light-induced self-poling effect possibly triggers the filling or repairing of trap states in the bulk. Second, it could be hypothesized that the surface recombination and carrier extraction were slowed down. However, TR results showed the opposite, as this process was found to be accelerated.

We employed transient spectroscopy in the reflection mode to measure the TR spectrum. The TR spectrum can be considered roughly proportional to the change of the real part of the refractive index, Δ*n*(ω), because the imaginary part (*k*) is trivial compared to *n* near the band edge. On the other hand, the TA spectrum provides the information of . Since Δ*n*(ω) and Δ*k*(ω) are linked through the Kramers–Kronig relationship, the Hilbert transform (HT) of TA can represent the TR spectrum, and the inverse Hilbert transform (iHT) of TR can represent the TA spectrum. The current state-of-the-art TR methodology only investigates a very shallow depth of the perovskite material (less than 30 nm), thereby being sensitive to the carrier kinetics at the interface.

Our TR measurements on both ETL–perovskite and perovskite–HTL interfaces are depicted in Figure 3b,c. Note that the TR dynamics were slightly accelerated by the light-soaking method at the perovskite–HTL interface. However, in the light-soaked device at the ETL–perovskite interface, the TR signal exhibited a significantly faster decay. The dynamics of interfacial carriers are primarily influenced by surface recombination and carrier diffusion/extraction into the bulk at the interface. In perovskites, the surface recombination layer is typically less than 5 nm,^63^ but our measurement has a penetration depth of around 20 nm, suggesting that the influence of diffusion/extraction is greater than that of surface recombination. The faster decay observed at the ETL–perovskite interface indicates a reduction in charge transfer resistance at the interface. Therefore, the light-soaking enhances carrier lifetime in the bulk and accelerates carrier extraction at the interfaces, particularly at the ETL–perovskite interface.

We further investigated the interfacial carrier dynamics at both the ETL–perovskite and perovskite–HTL junctions by employing TR spectroscopy. The penetration depth of the selected excitation wavelength is about 130 nm (Figure S7), and the state-of-the-art of TR probes only into a very shallow depth (less than 30 nm) of the perovskite, which is therefore sensitive to the interface carrier kinetics.^61^Figure 3b,c compares the carrier dynamics before and after light soaking, and both junctions show a faster decay in the light-soaked devices, especially at the ETL–perovskite interface. Interfacial carrier dynamics are mainly governed by surface recombination, carrier diffusion into the bulk, or charge transfer at the charge-separating interface. Because the KPFM study has shown a rise of the perovskite–HTL electric junction after light soaking, we attribute the faster decay here to the enhancement of the carrier transport process that is caused by electric drifting on the HTL side. However, a more pronounced reduction in the decay time is at the ETL side rather than at the HTL side, indicating a more reduced charge transfer resistance at the ETL–perovskite interface and mainly due to reduced surface recombination. These results complement the information provided in Figure 2c regarding the change of the electric field difference at the two junctions after light soaking.

**Figure 4:**
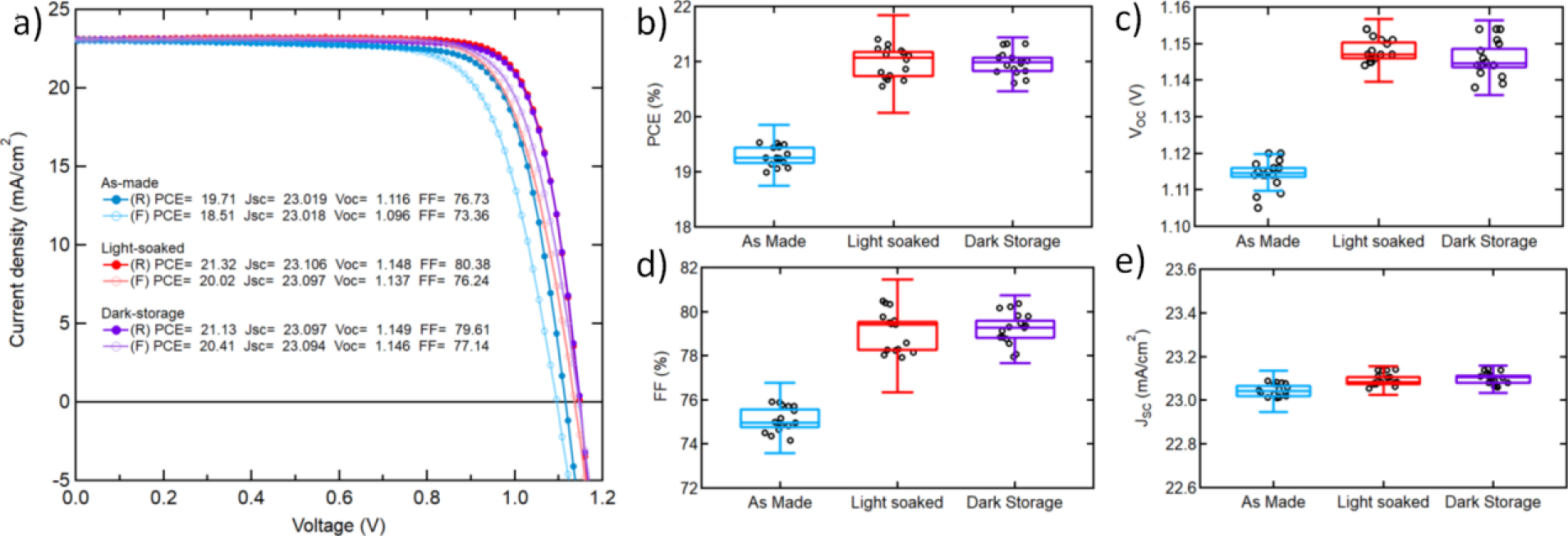
Photovoltaic performance of perovskite solar cells. (a) *J*–*V* curves of perovskite solar cells treated under different conditions. (b–e) Statistical distribution of photovoltaic parameters of 16 perovskite solar cells aged under different conditions.

We also investigated the impact of light soaking on the photovoltaic performance of PSCs and correlated the results to the changes in the electrostatic potential distribution and interfacial carrier dynamics across the device. Figure 4a shows the *J*–*V* curves of a representative device before and after light soaking for 30 min and subsequently followed by dark storage for 2 h. The as-made cell showed a PCE of 19.7% under reverse scan (18.5% under forward scan). After light soaking, the PCE increased to 21.3% under reverse scan (20.3% under forward scan), mainly due to the increased open-circuit voltage (*V*_OC_) and fill factor (FF). The detailed photovoltaic parameters are summarized in Table 1. The higher *V*_OC_ and FF indicate reduced recombination in the bulk and at the interface of the perovskite absorber layer, which is likely attributed to the enhanced perovskite junction quality as evidenced by the KPFM and ultrafast spectroscopy measurements. The stronger electric fields at the two interfaces of the perovskite absorber layer benefit the separation of photoexcited charge carriers, resulting in more efficient charge collection. The statistical results of a batch of as-made, light-soaked, and dark stored after light-soaking cells show the same trend (Figure 4b–e). The light-soaked cells retained almost the same efficiency after 2 h of storage in dark, indicating that the improvement in device performance is a persistent rather than a transient effect, consistent with the electrostatic potential distribution measured after storage in dark. A similar light-soaking effect on PSCs has also been reported by others and was also attributed to ion redistribution, which increases charge extraction and alleviates interface recombination.^49^ Our operando KPFM and ultrafast spectroscopy tools visualized and confirmed the effect, providing a possibility for detailed characterization of complex transient or reversible phenomena on the device level.

**Table 1:** Photovoltaic Performance Parameters of the Device under Different Treatments^a^

condition	*J*–*V* scan direction	PCE (%)	*V*_oc_ (V)	*J*_sc_ (mA/cm^2^)	FF (%)
as-made	rev	19.71	1.12	23.02	76.7
	fwd	18.51	1.10	23.02	73.4
light soaked 30 min	rev	20.80	1.15	23.09	78.3
	fwd	20.12	1.14	23.05	76.5
light soaked 2 h	rev	21.32	1.15	23.11	80.4
	fwd	20.31	1.15	23.09	76.5
leave in the dark for 2 h	rev	21.13	1.15	23.10	79.6
	fwd	20.41	1.15	23.10	77.1

a PCE = power conversion efficiency, *V*_oc_ = open-circuit voltage, and *J*_sc_ = short-circuit current.

## Conclusions

We applied cross-sectional KPFM and ultrafast spectroscopy techniques on the same batch of PSCs to study light-induced junction evolution and reveal the cell’s operational mechanisms. Our results showed that the pristine PSC operates as n–i–p with a main ETL–perovskite junction and a small perovskite–HTL junction. Additionally, a series of KPFM experiments performed on the same location showed reversible and irreversible electric junction evolution at both interfaces after light soaking, dark rest, and bias voltages. On the other hand, ultrafast spectroscopy results indicate that both junctions exhibit faster decay after light soaking, where the carrier dynamics changed more obviously at the ETL–perovskite interface possibly due to the reduced recombination rate. The combined KPFM and ultrafast spectroscopy, together with device performance, provide new routes for understanding charge-carrier transport and bulk/interface recombination in PSCs.

## Methods

### Solar Cell Fabrication and *J*–*V* Characterization

PSCs used in this study were fabricated in a nitrogen-filled glove box following the procedure reported previously [ref (50)]. The MA_0.63_FA_0.27_Cs_0.1_PbI_3_ precursor ink was prepared by dissolving 100 mg methylammonium iodide (MAI,Greatcell Solar Materials), 47 mg formamidinium iodide (FAI,Greatcell Solar Materials), 26 mg CsI (Sigma Aldrich), 461 mg PbI_2_ (Sigma Aldrich), and 9.2 mg Pb(SCN)_2_ (Sigma Aldrich) additive into a dimethylformamide (DMF)/dimethyl sulfoxide (DMSO) mixed solution (v/v = 630:71 μL). The precursor solution was stirred for 8 h and filtered with a 0.22 μm PTFE filter before spin coating. To fabricate PSCs, a 20 nm SnO_2_ layer was first deposited on FTO-coated glass substrates using plasma-enhanced atomic layer deposition. A C_60_-SAM self-assembly layer was then spin-coated as previously reported. The perovskite precursor ink was spin-coated on first at 500 rpm for 3 s and 4000 rpm for 60 s. Diethyl ether anti-solvent was dropped at ∼8 s of the second spin-process to promote rapid crystallization. After spin coating, the perovskite film was annealed at 65 °C for 2 min and then 100 °C for 5 min. 2,2′,7,7′-tetrakis(*N*,*N*′-di-*p*-methoxyphenylamine)-9,9′-spirobifluorene (Spiro-OMeTAD) was deposited on the perovskite film by spin coating at 3000 rpm for 60 s. The Spiro-OMeTAD solution was prepared by dissolving 72.3 mg Spiro-OMeTAD (Lumtec) in 1 mL chlorobenzene (Sigma Aldrich) with 28 μL 4-*tert*-butylpyridine (Sigma Aldrich), 18 μL Li-bis-(trifluoromethanesulfonyl) imide (Li-TFSI) (Sigma Aldrich) (520 mg/mL in acetonitrile), and 18 μL Co(II)-TFSI salt (Greatcell Solar Materials) (300 mg/mL in acetonitrile). Au or indium tin oxide (80 nm) was then deposited by thermal evaporation. The active area of the devices was 0.08 cm^2^. *J*–*V* characterizations were measured using a Keithley 2400 Sourcemeter under standard AM 1.5G illumination using a solar simulator (PV Measurements Inc.) calibrated to an output intensity of 100 mW/cm^2^. *J*–*V* curves were recorded in both forward and reverse voltage scans at a speed of 250 mV/s.

### Kelvin Probe Force Microscopy

KPFM was built based on a Bruker D5000 AFM. The Pt–Ir coated silicon probe (Nanosensor PPP-EFM) has two resonant peaks, one low-frequency peak (∼50 kHz) is used for topography, and the other higher frequency peak (∼350 kHz) is used for electrostatic force measurements. Topography and potential images were acquired at the same time, where the spatial resolution is 30 nm and the electrical resolution is 10 mV. The cell was cleaved directly without polishing or ion milling, but the cross-sectional surface is flat enough for KPFM scans. We applied forward and reverse bias voltages alternatively to minimize the ion migration effect. The scan on each bias voltage condition contains 1024 pixels in the fast-scan axis across the cell, and 32 lines in the slow-scan axis on the film direction, with a scan rate of 0.35 Hz that takes ∼90 s per scan. The scans were on the same location, judging by their topography. The cell was illuminated by a halide lamp at its maximum power, the light intensity is ∼0.5 Sun coming through the glass side. The forward and reverse bias voltages stressing utilized the KPFM setup. All sample preparation, light soaking, dark rest, biasing, and measurements were performed inside an Ar-filled glovebox with oxygen and water level <0.01 ppm.

### Ultrafast Transient Spectroscopy

Nanosecond TA spectra were collected using a commercial system (EOS) and a Coherent Libra Ti/sapphire laser, with an output of 800 nm at 1 kHz. The 800 nm beam was directed into a TOPAS optical parametric amplifier (OPA) to generate a pump pulse (∼150 fs) and was modulated at 500 Hz through an optical chopper to block every other laser pulse. The broadband EOS probe beam was electronically delayed with respect to the pump laser pulse. The femtosecond TR spectroscopy measurements were performed by a pump–probe spectrometer. The 800 nm fundamental laser pulse is generated by a Ti/sapphire amplifier. The pulse repetition rate is 1 kHz. The fundamental pulse is then split into two parts by a beam splitter. One part is sent to an OPA for pump generation at the selected wavelength. The pump is modulated at a frequency of 500 Hz and attenuated by neutral-density filter wheels. The other part of the fundamental pulse is focused into a sapphire crystal to generate a white-light continuum (1.55–2.7 eV) that is used as the probe. The probe pulses are delayed in time with respect to the pump pulses using a motorized translation stage mounted with a retro-reflecting mirror. The pump and probe are spatially overlapped on the surface of the sample. The incident angle for the probe is around 45 degree, while the pump beam incident the sample normally. The size of the focused spot at the sample position for the probe and pump beams is around 200 and 600 μm, respectively.

## Supporting Information Available

Electric potential profiling of the perovskite solar cell after light soaking, after dark rest, after applied +0.5 V forward bias voltage, applied −1 V reverse bias voltage, various potential profiling at 0 V, optical properties in MAFACsPbI_3_ devices before and after light soaking, and absorption coefficient and penetration depth of the MAFACsPbI_3_ perovskite (PDF)
