# Supplementary material for: Operando Characterizations of Light-Induced Junction Evolution in Perovskite Solar Cells
Source: ACS Appl Mater Interfaces. 2023 Apr 18;15(17):20909–16. doi: 10.1021/acsami.2c22801 (PMC10165603; doi:10.1021/acsami.2c22801)
Supplement: Supplementary file 1 — am2c22801_si_001.pdf [file am2c22801_si_001.pdf]

Supporting Information

Operando Characterizations of Light-Induced Junction Evolution in Perovskite Solar Cells

Chuanxiao Xiao,[1†*#] Yaxin Zhai,[2†#] Zhaoning Song,[3†] Kang Wang,[1#] Changlei Wang,[3#] Chun-Sheng Jiang,[1] Matthew C. Beard,[1] Yanfa Yan,[3] Mowafak Al-Jassim[1]

[1]National Renewable Energy Laboratory (NREL), Golden, CO 80401, USA

[2]Key Laboratory of Low-Dimensional Quantum Structures and Quantum Control of Ministry of Education, Department of Physics, Hunan Normal University, Changsha 410081, China

[3]The University of Toledo, Toledo, OH 43606, USA

†These authors contributed equally to this work.

[*]Correspondence to: Chuanxiao Xiao: cxiao@nimte.ac.cn

Present addresses:
[#]Chuanxiao Xiao: [1]Ningbo Institute of Materials Technology and Engineering, Chinese Academy of Sciences, Ningbo City, Zhejiang Province, 315201, China
[2]Ningbo New Materials Testing and Evaluation Center CO., Ltd, Ningbo City, Zhejiang Province, 315201, China

[#]Kang Wang: State Key Laboratory of Physical Chemistry of Solid Surfaces, College of Chemistry and Chemical Engineering, Xiamen University, Xiamen 361005, China

[#]Changlei Wang: School of Optoelectronic Science and Engineering & Collaborative Innovation Center of Suzhou Nano Science and Technology, Soochow University, Suzhou 215006, China

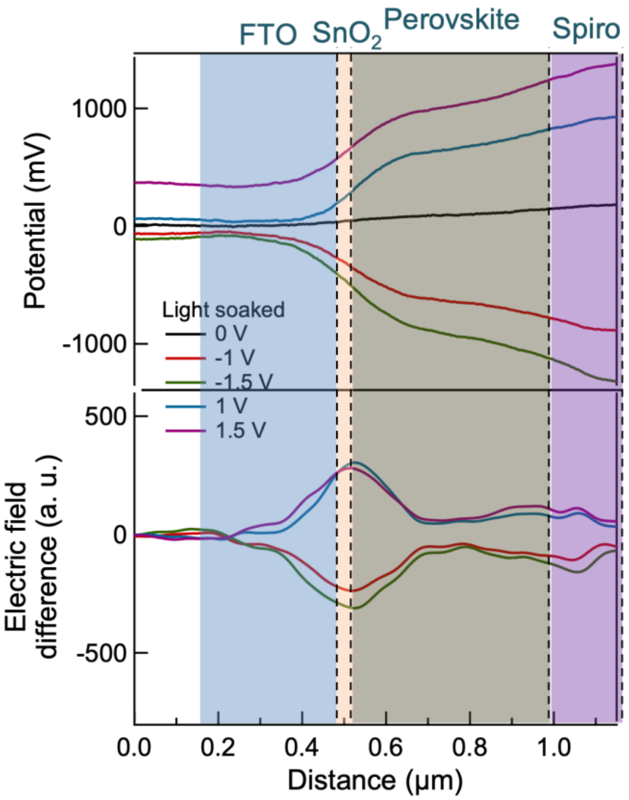

Figure S1. Electric potential profiling of the perovskite solar cell after light soaked. Top: potential profile average from 0 V, -1 V, 1 V, -1.5 V and 1.5 V bias voltages; Bottom: change in electric field, calculated by taking the first derivatives of the potential difference than the 0-V profile. The -1 V electric field difference profile is integrated in Figure 2c.

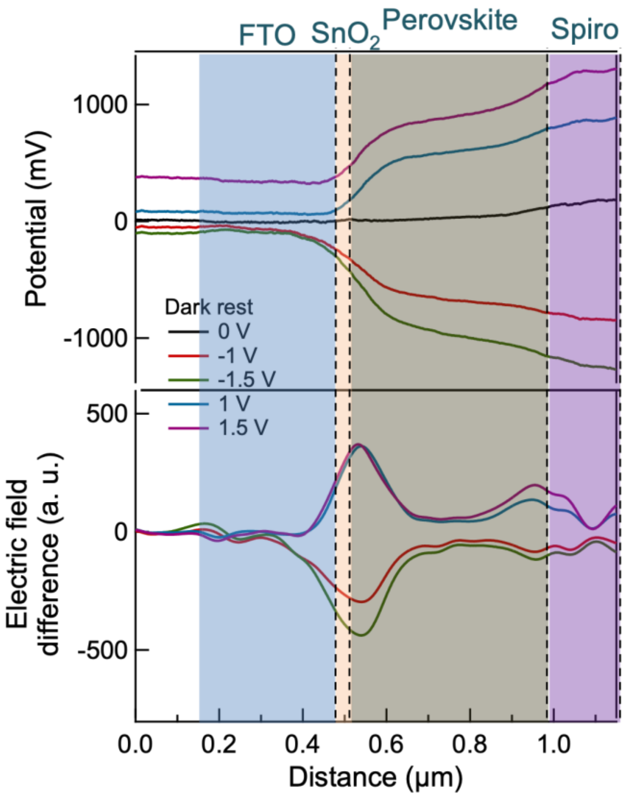

Figure S2. Electric potential profiling of the perovskite solar cell after dark rest. Top: potential profile average from 0 V, -1 V, 1 V, -1.5 V and 1.5 V bias voltages; Bottom: change in electric field, calculated by taking the first derivatives of the potential difference than the 0-V profile. The -1 V electric field difference profile is integrated in Figure 2c.

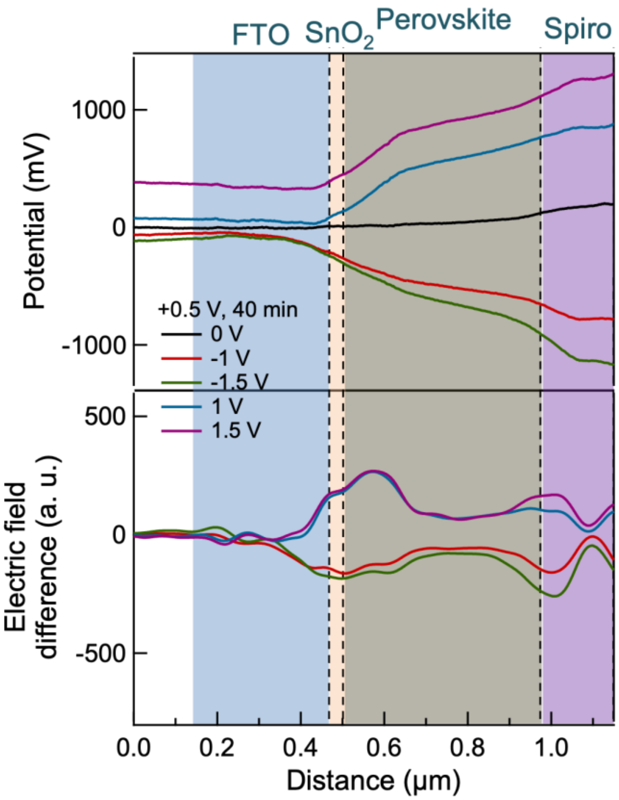

Figure S3. Electric potential profiling of the perovskite solar cell after applied +0.5 V forward bias voltage. Top: potential profile average from 0 V, -1 V, 1 V, -1.5 V, and 1.5 V bias voltages; Bottom: change in electric field, calculated by taking the first derivatives of the potential difference than the 0-V profile. The -1 V electric field difference profile is integrated in Figure 2c.

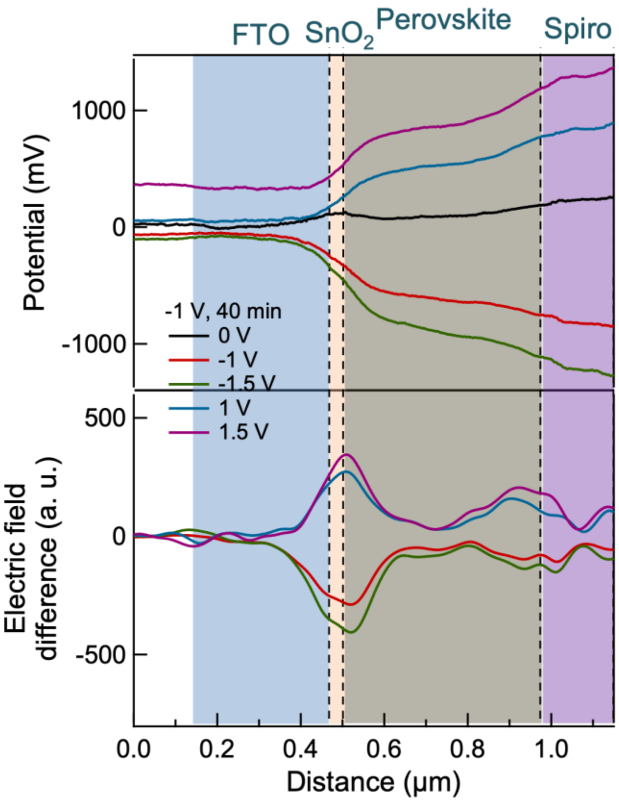

Figure S4. Electric potential profiling of the perovskite solar cell after applied -1 V reverse bias voltage. Top: potential profile average from 0 V, -1 V, 1 V, -1.5 V and 1.5 V bias voltages; Bottom: change in electric field, calculated by taking the first derivatives of the potential difference than the 0-V profile. The -1 V electric field difference profile is integrated in Figure 2c.

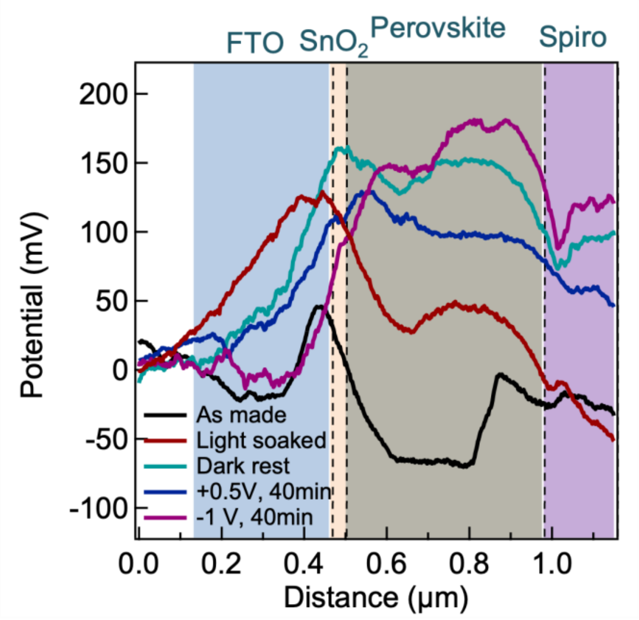

Figure S5. Potential profiling of the perovskite solar cell at 0-V that went through the process of (1) as-made, (2) light soaked, (3) after dark rest, (4) applied =0.5 V forward bias for 40minutes, and (5) applied -1 V reverse bias for 40minutes.

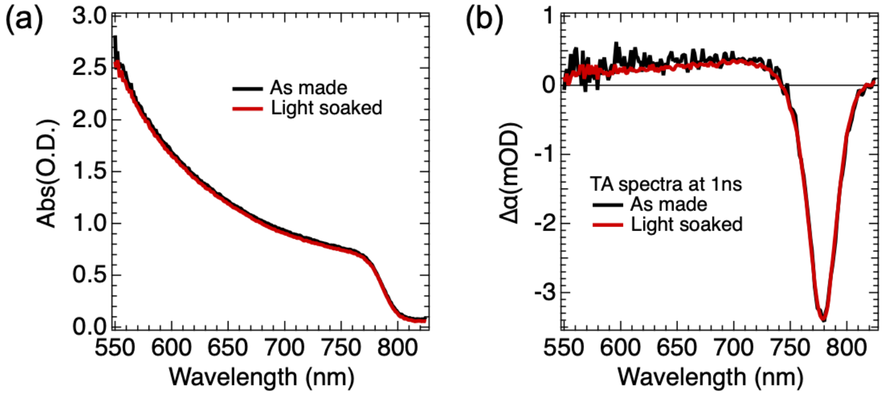

Figure S6. Optical properties in $MAFACsPbI_3$ devices before and after light soaking. (a) linear absorption spectra. (b) TA spectra at 1ns delay time. The light soaking process does not affect the linear and TA spectra.

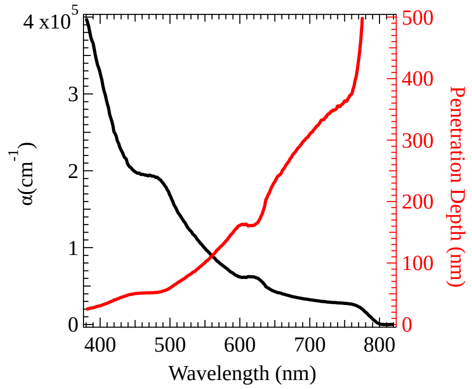

Figure S7. Absorption coefficient and penetration depth of the $FAMACsPbI_3$ perovskite.
